# Clinico-Epidemiological Profiles of COVID-19 Elderly Patients in Guwahati City, Assam, India: A Cross-Sectional Study

**DOI:** 10.7759/cureus.24043

**Published:** 2022-04-11

**Authors:** Priyanka Rana Patgiri, Vinoth Rajendran, Abdul B Ahmed

**Affiliations:** 1 Geriatrics, Regional Geriatric Centre, Gauhati Medical College & Hospital, Guwahati, IND; 2 Community Medicine & Family Medicine, All India Institute of Medical Sciences, Jodhpur, Jodhpur, IND; 3 General Medicine, Lakhimpur Medical College, Lakhimpur, IND

**Keywords:** risk factors of covid-19, symptoms of covid-19, india, clinical profile, elderly, covid-19

## Abstract

Background

As of November 14, 2021, coronavirus disease 2019 (COVID-19 has affected more than 3,44,00,000 individuals in India and resulted in more than 4,60,000 deaths in India.Symptoms of COVID-19 include cough, fever, dyspnea, diarrhea, fatigue, expectoration, myalgia, hemoptysis, abdominal pain, and anorexia.Associated comorbidities such as hypertension, diabetes, cardiovascular illness, and respiratory sicknesses influence the severity and prognosis of the COVID-19. Therefore, this study was conducted to determine the factors associated with the severity and outcome of elderly Indian people diagnosed with COVID-19.

Methodology

This hospital-based descriptive cross-sectional study was conducted among elderly patients with confirmed COVID-19 who were admitted to Gauhati Medical College Hospital from July 21, 2020, to January 15, 2021. The demographic data, exposure history, clinical symptoms and signs, underlying comorbidity, severity of COVID-19, and outcome data of each elderly patient were obtained and analyzed using SPSS software (Version 25.0, IBM Corp., Armonk, NY). The Fisher exact test, chi-square test, and binary logistic regression analysis were used for different study variables.

Result

A total of 165 hospitalized COVID-19 elderly patients were included in this study, and their mean age was 68.4 years. The most common symptoms were cough (34.5%), fever (28%), breathing difficulty (22%), weakness (13.1%), and chest pain (3.6%). Those with breathing difficulty (adjusted OR [aOR]: 7.293, 95% CI: 2.229-23.860, p=0.001), loose stool (aOR: 12.142, 95% CI: 1.052-140.209, p=0.045), hypertension (aOR: 2.703, 95% CI: 1.023-7.139, p=0.045), and severity of COVID-19 (aOR: 7.691, 95% CI: 2.870-20.607, P<0.001) had increased risk of poor outcome among the COVID-19 elderly. Being hypertensive (aOR: 2.474, 95% CI: 1.060-5.774, p=0.036) had an increased risk of severity of COVID-19.

Conclusion

The most common symptoms of COVID‐19 elderly patients were fever, cough, and breathing difficulty. In elderly COVID-19 patients, hypertension played a crucial role in determining the severity of COVID-19, whereas breathing difficulty, loose stool, hypertension, and moderate-to-severe COVID-19 elderly patients had a poor outcome.

## Introduction

The outbreak of coronavirus disease 2019 (COVID-19), named the severe acute respiratory syndrome coronavirus 2 (SARS-CoV-2) initially, started in December 2019 in Wuhan, China. Later, it has quickly turned into a worldwide pandemic [1]. The first patient with confirmed COVID-19 in India was identified to be from Kerala, India, on January 27, 2020 [2]. Shortly afterward, the infection spread rapidly throughout the country. As of November 14, 2021, COVID-19 has affected more than 3,44,00,000 individuals in India and has resulted in more than 4,60,000 deaths in India [3].

According to the meta-analysis conducted by He et al., COVID-19 infection can be diagnosed based on symptoms such as on cough, fever, dyspnea, diarrhea, fatigue, expectoration, myalgia, hemoptysis, abdominal pain, and anorexia [4]. The study conducted by Zheng et al. stated that male sex and age more than 65 may pose a greater risk of developing critical or mortal conditions among the elderly who were diagnosed with COVID-19 [5]. Furthermore, men are associated with unhealthy lifestyle habits such as smoking and underlying disorders, both of which have been linked to an elevated risk of significant COVID-19 related diseases [5,6]. The highest number of comorbidities has been seen in severe cases of COVID-19, recommending that chronic diseases are probably the factors for adverse clinical outcomes. Many studies revealed that comorbidities, such as hypertension, chronic obstructive pulmonary disease, diabetes, and cardiovascular disease, were risk factors for severity and mortality of COVID-19 patients [4,7,8].

Diabetes mellitus has been associated with a higher risk of poor outcome and mortality among COVID-19 participants in several studies [7,9,10]. Individuals with diabetes are characterized by pulmonary dysfunction because of diminished lung volume, reduced pulmonary diffusing capacity, ventilation control, bronchomotor tone, and noradrenergic innervation impairment [11]. Diabetics are more susceptible to disease because of the related lymphopenia and exaggerated inflammatory response related to an increased renin-angiotensin system (RAS) activation in various tissues [12]. Furthermore, the glycometabolic control and the type of drug used for diabetic treatment also play an important role in determining the outcome of COVID-19 [9,13,14].

In patients with SARS-CoV-2 infection, hypertension has been identified as an independent risk factor for increased death and morbidity [7,9,10]. The specific underlying mechanisms that relate hypertension to worse COVID-19 outcomes are still not clear. COVID-19 attacks the alveolar epithelial cells through angiotensin-converting enzyme 2 (ACE2). Individuals who consume antihypertensive medications such as ACE inhibitors (ACEis) and angiotensin receptor blockers undergoing renin-angiotensin-aldosterone inhibition may be related to enhanced ACE2 expression at the cell surface, providing SARS-CoV-2 with a larger number of “anchors” for infecting cells, and the patients thus become more susceptible to COVID-19 infection and adverse consequences [15,16].

In comparison to non-severe patients, severe cases were more likely to develop acute respiratory distress syndrome (ARDS), septic shock, metabolic acidosis, and coagulopathy that were difficult to correct. Furthermore, severe patients were more likely to experience kidney, heart, and other organ damage, as well as multiple organ failures [17,18]. Because severe COVID-19 cases are more likely to develop ARDS, septic shock, or metabolic acidosis, it is now widely accepted that severe COVID-19 cases have a greater fatality rate than mild ones. As a result, it is critical to identify between severe and mild individuals early on.

Healthcare providers must identify the risk factors and underlying diseases of COVID-19 patients. By taking into account the risk factors for COVID-19 critical cases, properly allocating medical resources, identifying severe patients in the early stages of the disease, and devising an appropriate treatment plan, the mortality rate can be reduced and treatment effectiveness can be improved [19,20]. Therefore, it is important to determine the factors associated with the severity and outcome in elderly Indian people diagnosed with coronavirus who were hospitalized because of the disease, which will provide help in controlling the epidemic disease.

## Materials and methods

This research was a hospital-based descriptive cross-sectional study of elderly patients with confirmed COVID-19 who were admitted to COVID Hospital 3 of Gauhati Medical College Hospital from July 21, 2020, to January 15, 2021, affiliated to the Gauhati Medical College and Hospital (GMCH) located in Guwahati city, India, which is one of the tertiary care centers. The inclusion criteria were the elderly patients above the age of 60 years of both sexes who were willing to participate in the study and elderly patients who were confirmed to have been infected by the novel coronavirus disease either by rapid antigen test (RAT) or reverse transcriptase-polymerase chain reaction (RT-PCR) on throat and nose swab samples. All the elderly meeting the eligibility criteria were included in the study. Therefore, sample size calculation was not required in this study.

A pre-designed and pretested interview schedule was developed using the most recent available information about COVID-19 from PubMed and the Centers for Disease Control and Prevention (CDC). The demographic data, exposure history, clinical symptoms and signs, underlying comorbidity, severity of COVID-19 disease, and outcome data of each patient were obtained using standardized data collection forms. All the data collected were checked by a team of experienced doctors and entered into a computer database. Elderly patients with missing data on the characteristics studied or with unknown medical records were excluded. Elderly with a known medical history of bronchial asthma and on medication as evidenced by medical records were taken as asthmatic.

The CDC diagnostic criteria [21] were used to diagnose diabetes, which was defined as fasting plasma glucose > 126 mg/dL (HbA1C test > 6.5%) or when the patients were on anti-diabetics as evidenced by medical records. Hypertension was defined using CDC guidelines after taking two readings, one to two minutes apart, and, on average, the readings were taken to classify as hypertensive patients or when the patients were on anti-hypertensives as evidenced by medical records [22]. Elderly without shortness of breath or hypoxia (normal saturation, SpO_2_ > 93%) were classified as mild COVID-19 cases, whereas those with the presence of clinical features of dyspnea and/or hypoxia, fever, and cough, including SpO2 ≤ 93% on room air and respiratory rate more or equal to 24 breaths per minute were classified as moderate-to-severe COVID-19 cases [23]. All clinical outcomes of elderly patients were presented after completing the hospital period at the end of the study. Operational definitions were made for classifying the outcome of the COVID-19 cases. The elderly who tested negative and were discharged from the hospital within 10 days were classified as having good outcomes, whereas patients who were shifted to ICU, those who died, or those who were not discharged after day 10 were classified as having poor outcomes. The ethical clearance was taken from the Institutional Ethics Committee of GMCH.

Statistical analysis

The continuous variables were presented as mean ± standard deviation (SD) and the categorical variables were expressed as frequencies and percentages (%). The Fisher exact test or chi-square test was applied to compare categorical variables as appropriate. Potential confounders were chosen based on data from previous studies. Adjusted confounders in our study included age, gender, diabetes, hypertension, asthma, and severity of COVID-19. The adjusted binary logistic regression analysis has been used for the analysis of the severity and outcome of COVID-19. The data were analyzed using SPSS software (Version 25.0, IBM Corp., Armonk, NY). For all the statistical analyses, a p-value of <0.05 was considered statistically significant.

## Results

A total of 165 hospitalized COVID-19 elderly patients were included in this cross-sectional study, and their mean age was 68.4 years. The distribution of respondents based on the clinico-epidemiological profile of COVID is shown in Table 1. The majority (125, 75.8%) of patients were males, and the maximum number of elderly patients were in the age group of 60-74 years (80.6%). The highest number of elderly patients admitted was from Kamrup (Metro) district, which indicated an urban population. Most of the elderly were asymptomatic (69%) on the date of admission. The most commonly seen symptoms were cough (35.2%) and fever (28.5%), followed by breathing difficulty (22.4%), weakness (13.3%), and chest pain (3.6%). Among the elderly, 37.6% and 24.2% were hypertensive and diabetic, respectively.

**Table 1:** Distribution of respondents based on the clinico-epidemiological profile of COVID-19.

Clinico-Epidemiological Profile	Number	Percentage
Age (mean ± SD)	68.4 ± 6.9	-
Sex		
Female	40	24.2%
Male	125	75.8%
Clinical profile		
Cough	58	35.2%
Fever	47	28.5%
Breathing difficulty	37	22.4%
Weakness	22	13.3%
Chest pain	6	3.6%
Abdominal pain	4	2.4%
Loose stool	4	2.4%
Chronic morbidities		
Hypertension	62	37.6%
Diabetes mellitus	40	24.2%
Asthma	3	1.8%
Outcome		
Good	126	76.4%
Poor	39	23.6%
Severity		
Mild cases	121	73.3%
Moderate-to-severe cases	44	26.7%

The relationship of the clinico-epidemiological profile with the severity of COVID-19 is given in Table 2. The mean ages of the elderly with mild cases and moderate-to-severe cases of COVID-19 were 68.3 and 68.7, respectively. Of the total elderly who had breathing problems, 37.8% developed a moderate-to-severe form of COVID-19 disease. Among the elderly with hypertension, 37.1% were found to be having moderate-to-severe cases of COVID-19.

**Table 2:** Relationship of the clinico-epidemiological profile with the severity of COVID patients.

Clinico-epidemiological profile	Severity			
	Mild cases (n=121)		Moderate-to-severe cases (n=44)	
	Column, n (%)	Row (%)	Column, n (%)	Row (%)
Age (mean ± SD)	68.3 ± 0.6	-	68.7 ± 1.0	-
Sex				
Female	33 (27.3%)	82.5%	7 (15.9%)	17.5%
Male	88 (72.7%)	70.4%	37 (84.1%)	29.6%
Clinical profile				
Fever	36 (29.8%)	76.6%	11 (25.0%)	23.4%
Cough	40 (33.1%)	69.0%	18 (40.9%)	31.0%
Breathing difficulty	23 (19.0%)	62.2%	14 (31.8%)	37.8%
Chest pain	4 (3.3%)	66.7%	2 (4.5%)	33.3%
Weakness	16 (13.2%)	72.7%	6 (13.6%)	27.3%
Abdominal pain	3 (2.5%)	75.0%	1 (2.3%)	25.0%
Loose stool	3 (2.5%)	75.0%	1 (2.3%)	25.0%
Chronic morbidities				
Hypertension	39 (32.2%)	62.9%	23 (52.3%)	37.1%
Diabetes mellitus	31 (25.6%)	77.5%	9 (20.5%)	22.5%
Asthma	2 (1.7%)	66.7%	1 (2.3%)	33.3%

The relationship of the clinico-epidemiological profile with the outcome of COVID is given in Table 3. The mean age in good and poor outcomes of COVID-19 were 67.89 and 69.69, respectively. Among the elderly with diabetes mellitus and hypertension, 22.5% and 27.4% were found to be having poor outcomes, respectively. Moderate-to-severe cases of COVID-19 elderly had a comparatively poor outcome (47.7%) than the mild cases of COVID-19, where only 14.9% had poor outcomes.

**Table 3:** Relationship of the clinico-epidemiological profile with the outcome of COVID patients.

Clinico-epidemiological profile	Outcome			
	Good (n=126)		Poor (n=39)	
	Column, n (%)	Row (%)	Column, n (%)	Row (%)
Age (mean ± SD)	68.0 ± 0.6	-	69.7 ± 1.2	-
Sex				
Female	34 (27.0%)	85.0%	6 (15.4%)	15.0%
Male	92 (73.0%)	73.6%	33 (84.6%)	26.4%
Clinical profile				
Fever	37 (29.4%)	78.7%	10 (25.6%)	21.3%
Cough	45 (35.7%)	77.6%	13 (33.3%)	22.4%
Breathing difficulty	22 (17.5%)	59.5%	15 (38.5%)	40.5%
Chest pain	5 (4.0%)	83.3%	1 (2.6%)	16.7%
Weakness	19 (15.1%)	86.4%	3 (7.7%)	13.6%
Abdominal pain	3 (2.4%)	75.0%	1 (2.6%)	25.0%
Loose stool	2 (1.6%)	50.0%	2 (5.1%)	50.0%
Chronic morbidities				
Hypertension	45 (35.7%)	72.6%	17 (43.6%)	27.4%
Diabetes mellitus	31 (24.6%)	77.5%	9 (23.1%)	22.5%
Asthma	2 (1.6%)	66.7%	1 (2.6%)	33.3%
Severity				
Mild cases	103 (81.7%)	85.1%	18 (46.2%)	14.9%
Moderate-to-severe cases	23 (18.3%)	52.3%	21 (53.8%)	47.7%

The association of clinico-epidemiological variables with the severity of COVID-19 among the elderly is shown in Table 4. The crude odds ratio and adjusted odds ratio (aOR) were calculated, and it was seen that being hypertensive (aOR: 2.474, 95% CI: 1.060-5.774, p=0.036) had increased risk of severity of COVID-19. There was no significant difference between non-severe and severe patients in terms of age, sex, fever, cough, breathing difficulty, chest pain, weakness, abdominal pain, loose stool, diabetes, and asthma.

**Table 4:** Association of clinico-epidemiological variables with the severity of COVID-19 among elderly.

Clinico-epidemiological variables	Crude odds ratio (95% confidence interval)	p-Value	Adjusted odds ratio (95% confidence interval)	p-Value
Age	1.01 (0.96-1.06)	0.74	0.80 (0.32-1.99)	0.63
Sex	1.98 (0.81-4.88)	0.13	0.49 (0.19-1.28)	0.15
Asymptomatic	0.43 (0.19-1.02)	0.05	3.19 (0.99-10.21)	0.05
Fever	0.79 (0.36-1.73)	0.55	1.72 (0.68-4.37)	0.25
Cough	1.40 (0.69-2.85)	0.35	1.10 (0.46-2.67)	0.83
Breathing difficulty	1.99 (0.91-4.34)	0.08	0.76 (0.31-1.90)	0.56
Chest pain	1.39 (0.25-7.89)	0.51	1.47 (0.23-9.44)	0.68
Weakness	1.04 (0.38-2.84)	0.95	1.01 (0.34-3.01)	0.99
Abdominal pain	0.92 (0.09-9.03)	0.71	1.59 (0.14-17.85)	0.71
Loose stool	0.92 (0.09-9.03)	0.71	1.57 (0.13-18.59)	0.72
Hypertension	0.43 (0.22-0.88)	0.03	2.47 (1.06-5.77)	0.04
Diabetes	0.75 (0.32-1.73)	0.49	1.08 (0.43-2.74)	0.87
Asthma	2.83 (0.39-20.75)	0.29	0.58 (0.07-5.26)	0.63

The association of clinico-epidemiological variables with the outcome of COVID-19 among the elderly is shown in Table 5. The crude odds ratio and aORs were calculated to identify the various clinico-epidemiological factors influencing the outcome of COVID-19 among the elderly. It has been found that those with breathing difficulty (aOR: 7.293, 95% CI: 2.229-23.860, p=0.001), loose stool (aOR: 12.142, 95% CI: 1.052-140.209, P= 0.045), hypertension (aOR: 2.703, 95% CI: 1.023-7.139, p=0.045 ), and severity of COVID-19 (aOR: 7.691, 95% CI: 2.870-20.607, p<0.001) had an increased risk of poor outcome among the COVID-19 elderly. There was a borderline statistically significant association of age with the outcome of COVID-19. There was no significant difference between the patients regarding the outcome in terms of sex, fever, cough, chest pain, weakness, abdominal pain, diabetes, and asthma.

**Table 5:** Association of clinico-epidemiological variables with outcome of COVID-19 among elderly.

Clinico-epidemiological variables	Crude odds ratio (95% confidence interval)	p-Value	Adjusted odds ratio (95% confidence interval)	p-Value
Age	1.04 (0.98-1.09)	0.18	2.74 (0.99-7.50)	0.05
Sex	2.03 (0.78-5.28)	0.14	1.51 (0.51-4.53)	0.46
Asymptomatic	0.91 (0.41-2.01)	0.82	4.22 (0.94-18.99)	0.06
Fever	0.83 (0.37-1.87)	0.65	1.97 (0.61-6.35)	0.26
Cough	0.90 (0.42-1.92)	0.79	0.75 (0.24-2.28)	0.61
Breathing difficulty	2.96 (1.34-6.53)	0.01	7.29 (2.23-23.86)	0.00
Chest pain	0.64 (0.07-5.62)	1.00	0.46 (0.03-7.67)	0.59
Weakness	0.47 (0.13-1.68)	0.24	0.66 (0.15-2.80)	0.57
Abdominal pain	1.08 (0.11-10.68)	1.00	1.92 (0.15-25.25)	0.62
Loose stool	3.35 (0.46-24.62)	0.24	12.14 (1.05-140.21)	0.05
Hypertension	1.39 (0.67-2.89)	0.38	2.70 (1.02-7.14)	0.05
Diabetes	0.92 (0.39-2.15)	0.85	1.04 (0.38-2.89)	0.94
Asthma	3.35 (0.46-24.62)	0.24	10.81 (0.74-157.47)	0.08
Severity	5.23 (2.41-11.34)	0.00	7.69 (2.87-20.61)	0.00

## Discussion

The present research was a descriptive cross-sectional study of 165 hospitalized elderly patients with COVID-19 that were analyzed in terms of demographic and clinical characteristics, and outcomes. The mean age of the elderly was 68.39 years, which ranged from 60 to 89 years, and the maximum number of elderly patients was in the age group of 60-74 years (80.6%). Similarly, a high prevalence of COVID-positive elderly was found in this age group (mean ± SD: 60.9±8.2), as reported in previous studies [9]. In the current study, majority of the hospitalized elderly were men (75.8%), a finding similar to the study conducted by Fathi et al., where they found that SARS-CoV-2 affects men (54.92%, 95% CI: 52.92-56.92, I^2^=96.53%) more than women (45.12%, 95% CI: 43.12-47.12, I^2^=96.55%) [7]. This could be due to the protection provided by the X chromosome and sex hormones, both of which have a role in the COVID-19 distribution among the elderly. In addition, men go out more frequently and tend to adopt harmful lifestyle habits such as smoking, alcohol consumption, and underlying disorders, which could lead to an increased risk of significant COVID-19 related diseases [5,6]. The borderline statistically significant association of age with the outcome of COVID-19 is similar to the other studies, where they showed that the mortality rate progressively increased with age [9,24].

According to findings in the current study, cough (35.2%), fever (28.5%), and difficulty in breathing (22.4%) were the most common symptoms in the elderly patients, whereas abdominal pain (2.4%) and loose stool (2.4%) were uncommon, findings consistent with previous studies [7,9,19]. However, the proportion of these symptoms are comparatively lower in the current study than in the other studies, and this may be due to different strategies followed by different regions/areas for screening of the suspected or contacts of COVID-19 cases. In the region where contact tracing of COVID-19 cases is intensified, many asymptomatic cases may be identified before the development of any symptoms. These symptoms will help in the early identification of the COVID-19 cases in the community. Early diagnosis and isolation of patients are critical in avoiding the spread of infectious diseases, but since SARS-Cov-2 includes additional transmission routes (e.g., fomites), controlling its rapid global spread is much more difficult. As a result, the best way to protect the population from infection is to improve personal protection and communal hygiene management [19,20].

In the current study, elderly patients with breathing difficulty (aOR=7.293, 95% CI: 2.229-23.860), severity of COVID-19 (aOR=7.691, 95% CI: 2.870-20.607), and loose stool (aOR=12.142, 95% CI: 1.052-140.209) showed poor outcome than patients without these symptoms, which could be significantly related to a higher incidence of complications and coexistence with other commodities. The meta-regression analysis of the study conducted by Corona et al. showed that the mortality rate was directly related to subjective dyspnea and fatigue as well to respiratory rate. Conversely, no association between a higher mortality rate and other symptoms such as fever, cough, sore throat, or gastrointestinal problems (including diarrhea, nausea, or vomiting) was observed [9]. The association of loose stool with poor outcome in the current study is contrary to that in Corona et al.’s study, and this may be because they have seen association only with mortality rate, whereas in the current study, mortality rates and shifting to the ICU are taken as poor outcome.

The findings in the current study showed that hypertension was found in 43.6% of elderly patients with poor outcomes in comparison to 35.7% of elderly patients with a good outcome, and 52.3% of those with severe COVID-19 in comparison to 32.2% of non-severe COVID-19. Our findings are also in line with the results of the meta-analysis conducted by Du et al., in which hypertension was found in 37.0% (95% CI: 0.27-0.47) of critical COVID-19 patients compared to 18.0% (95% CI: 0.14-0.23) of non-critical COVID-19 patients, and 46.0% (95% CI: 0.37-0.55) of those who died compared to 22.0% (95% CI: 0.16-0.28) of survivors [8]. Based on our results of the current study, individuals with hypertension should receive serious attention for treatments and vaccinations useful for SARS-CoV-2 as they tend to develop severe COVID-19 (aOR=2.474, 95% CI: 1.060-5.774) and have a poor outcome (aOR=2.703, 95% CI: 1.023-7.139). Similarly, pooled results based on aOR for patients with hypertension showed a 1.82-fold greater risk of critical COVID-19 (aOR: 1.82, 95% CI: 1.19 - 2.77, p=0.005) and a 2.17-fold higher risk of COVID-19 mortality (aOR: 2.17, 95% CI: 1.67-2.82, p=0.001) [8].

The association between the outcome and severity of COVID-19 with diabetes mellitus was not found to be significant in the current study. It is contrary to the findings of the various studies, where there is a significant relationship between them [7,9,10]. In COVID-19 patients, diabetes was associated with a two-fold increased risk of poor outcome and mortality [25]. This may be due to the difference in the glycometabolic control and the type of drug that has been used for diabetic treatment, which could determine the outcome of COVID-19 [9,13,14]. Hence, further studies are required, which will include all these factors.

## Conclusions

In summary, current evidence shows that the most commonly experienced symptoms of COVID‐19 elderly patients were fever, cough, and breathing difficulty. In elderly COVID-19 patients, associated morbidities, and mostly hypertension, play a crucial role in determining the severity of COVID-19 hospitalized patients. Patients with increasing age, breathing difficulty, loose stool, hypertension, and moderate-to-severe COVID-19 patients had a poor outcome.
